# Phosphorylation-Dependent Targeting of *Tetrahymena* HP1 to Condensed Chromatin

**DOI:** 10.1128/mSphere.00142-16

**Published:** 2016-08-24

**Authors:** Katerina Yale, Alan J. Tackett, Monica Neuman, Emily Bulley, Brian T. Chait, Emily Wiley

**Affiliations:** a W. M. Keck Science Center of Claremont McKenna, Pitzer, and Scripps Colleges, Claremont, California, USA; b Laboratory of Mass Spectrometry and Gaseous Ion Chemistry, The Rockefeller University, New York, New York, USA; Academia Sinica

**Keywords:** HP1, *Tetrahymena*, chromatin, chromodomain, ciliates, heterochromatin, protein phosphorylation

## Abstract

The evolutionarily conserved proteins related to heterochromatin protein 1 (HP1), originally described in *Drosophila*, are well known for their roles in heterochromatin assembly and gene silencing. Targeting of HP1 proteins to specific chromatin locales is mediated, at least in part, by the HP1 chromodomain, which binds to histone H3 methylated at lysine 9 that marks condensed regions of the genome. Mechanisms that regulate HP1 targeting are emerging from studies with yeast and metazoans and point to roles for posttranslational modifications. Here, we report that modifications of an HP1 homolog (Hhp1) in the ciliate model *Tetrahymena thermophila* correlated with the physiological state and with nuclear differentiation events involving the restructuring of chromatin. Results support the model in which Hhp1 chromodomain binds lysine 27-methylated histone H3, and we show that colocalization with this histone mark depends on phosphorylation at a single Cdc2/Cdk1 kinase site in the “hinge region” adjacent to the chromodomain. These findings help elucidate important functional roles of reversible posttranslational modifications of proteins in the HP1 family, in this case, regulating the targeting of a ciliate HP1 to chromatin regions marked with methylated H3 lysine 27.

**IMPORTANCE** Compacting the genome to various degrees influences processes that use DNA as a template, such as gene transcription and replication. This project was aimed at learning more about the cellular mechanisms that control genome compaction. Posttranslational modifications of proteins involved in genome condensation are emerging as potentially important points of regulation. To help elucidate protein modifications and how they affect the function of condensation proteins, we investigated the phosphorylation of the chromatin protein called Hhp1 in the ciliated protozoan *Tetrahymena thermophila*. This is one of the first functional investigations of these modifications of a nonhistone chromatin condensation protein that acts on the ciliate genome, and discoveries will aid in identifying common, evolutionarily conserved strategies that control the dynamic compaction of genomes.

## Abstract (precis)

Compacting the genome to various degrees influences processes that use DNA as a template, such as gene transcription and replication. This project was aimed at learning more about the cellular mechanisms that control genome compaction. Posttranslational modifications of proteins involved in genome condensation are emerging as potentially important points of regulation. To help elucidate protein modifications and how they affect the function of condensation proteins, we investigated the phosphorylation of the chromatin protein called Hhp1 in the ciliated protozoan *Tetrahymena thermophila*. This is one of the first functional investigations of these modifications of a nonhistone chromatin condensation protein that acts on the ciliate genome, and discoveries will aid in identifying common, evolutionarily conserved strategies that control the dynamic compaction of genomes.

## INTRODUCTION

The organization of DNA into higher-order chromatin domains is critical for the regulation of eukaryotic genes throughout the cell cycle and complex developmental processes. Assembly and maintenance of euchromatin and heterochromatin largely depend on specific posttranslational histone modifications, which recruit specific “effector proteins” that influence those domains. How the binding of key effectors to their target genomic regions is modulated remains a central question that underlies deeper understandings of chromatin-based gene regulation mechanisms.

Proteins in the heterochromatin protein 1 (HP1) family are well known as key effectors of repressive heterochromatin domains in yeast, protozoa, insects, plants, and mammals. First discovered in *Drosophila melanogaster*, HP1 proteins are defined as having a chromodomain (CD) at the N terminus and a C-terminal “chromoshadow” domain (CSD), linked by a “hinge region” between the two domains (1, 2). The CD and hinge region cooperate to bind the chromatin template, while the CSD serves as an interface for homodimerization and interactions with other proteins (reviewed in references [Bibr B3][Bibr B4][Bibr B5]). Most of the organisms examined so far possess multiple paralogs with this domain architecture. For example, humans, mice, and *Xenopus* express three, *Drosophila* expresses at least five, *Caenorhabditis elegans* and fission yeast express two (4, 6), and there are at least three in the ciliate *Tetrahymena* (E. Wiley, unpublished data). It is not unusual for paralogs to have distinct nuclear functions and locales. Although known best for roles in heterochromatin-mediated repression, some HP1 homologs have roles in DNA repair, replication, RNA splicing, telomere maintenance, and transcriptional activation and elongation (reviewed in reference 7). In some cases, a single HP1 protein may have multiple nuclear functions. This versatility in function depends, in part, on specific interactions with other factors, primarily via the hinge region and CSD (4, 7). Interactions between HP1 and chromatin have central roles in chromatin biology, and understanding how they are regulated has been an important pursuit.

Targeting of HP1 proteins to heterochromatin locales is specified in part by the CD, which binds methylated lysine 9 on histone H3 (H3K9Me), a feature of constitutive heterochromatin (8, 9). CDs are also present on repressor proteins related to *Drosophila* Polycomb (Pc) that bind to genomic locations marked with lysine 27-methylated histone H3 (H3K27Me_3_) (10 – 13). Although they are similar in sequence and structure, high-resolution structural comparisons have identified key amino acids that specify the differential interactions of CDs from HP1 and Pc proteins with methylated lysine 9 or lysine 27, respectively (12, 14).

Proteins containing CDs are often posttranslationally modified, and some modifications have been shown to regulate biological functions of these proteins through interactions with other proteins and/or by modulating affinities for target sites. On HP1 proteins, the predominant phosphorylation modifications are the best studied. Phosphorylation of *Drosophila* HP1a (dHP1a) in the N terminus, C terminus, and hinge regions correlates with heterochromatin formation during development and is essential for the gene-silencing activity of pericentric heterochromatin (15, 16). Other studies have revealed that phosphorylation of serine residues in the CSD of dHP1 decreases its homodimerization but increases its binding with other protein partners that modify its function (17). In mammalian cells, serine phosphorylation within an acidic N-terminal extension (NTE) of the CD increased HP1’s affinity for H3K9Me_3_ peptides *in vitro* (18) and increased its binding specificity for H3K9me-marked nucleosomes (19), while phosphorylation at some sites farther from the CD had no effect on the ability of a human HP1 paralog to bind to methylated chromatin (20). The variability in the location and number of phosphoryl modifications on HP1 homologs between and within species indicates that there is more to be learned about patterns that relate to function by studying these proteins in a broad range of organisms.

The ciliated protozoan *Tetrahymena thermophila* is a useful model for examining the regulation of chromatin proteins related to chromatin dynamics. *Tetrahymena* cells exhibit nuclear dimorphism: each cell contains a transcriptionally active macronucleus and a transcriptionally silent germline micronucleus composed entirely of highly condensed chromatin (21). Chromatin-restructuring events underlying the differentiation and development of these functionally distinct nuclei can be easily synchronized in a population of cells. An HP1 homolog (called Hhp1) that localizes exclusively to the macronucleus (not the silent micronucleus) was found enriched in the highly condensed chromatin regions call chromatin bodies (22, 23), which are marked with H3K27Me_3_ (24). The phosphorylation of Hhp1 varies with the physiological state: cell starvation induces hyperphosphorylation that correlates with the assembly of more chromatin into condensed chromatin bodies, a process that requires Hhp1 (22). The present study explored the requirements for the targeting of Hhp1 to condensed chromatin bodies. We report that the CD of Hhp1 possesses sequence features common to Pc-type CDs that facilitate recognition of the H3K27Me_3_ mark and that Hhp1 colocalizes with macronuclear histone H3K27Me_3_ *in vivo* in a manner dependent on the aromatic methyl-lysine binding “cage” within the CD. Two sites of phosphorylation common to multiple isoforms were located within the hinge region. The most abundant phosphorylation, at threonine 64 (T64), appeared to be required for Hhp1 concentration on H3K27Me_3_-marked chromatin bodies, suggesting that reversible phosphorylation adjacent to the CD can serve as a mechanism for regulating binding to the H3K27Me_3_ modification.

## RESULTS

*Tetrahymena* Hhp1 was originally identified as a member of the HP1 family by virtue of its amino-terminal CD and carboxy-terminal CSD. However, HP1 homologs typically bind histone H3K9-methylated chromatin, which is absent from *Tetrahymena* macronuclei, where Hhp1 resides (25). Instead, macronuclear chromatin contains trimethylated histone H3K27, a mark that is enriched in the highly condensed, repressive “chromatin body” structures within the macronucleus, where Hhp1 concentrates (23, 24).

**FIG 1:**
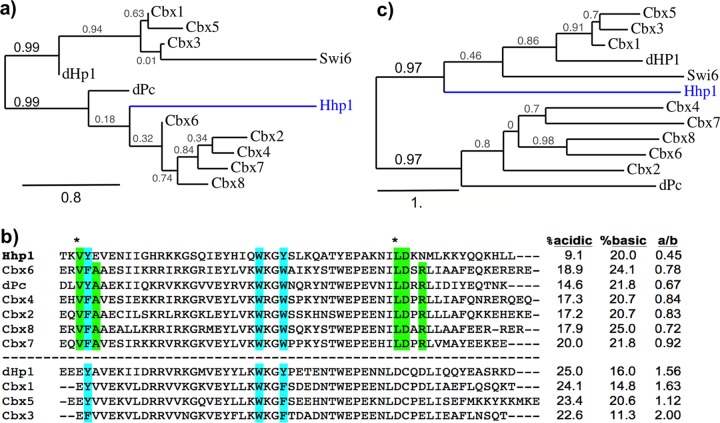
Hhp1 CD is more similar to Pc-type than HP1-type CDs. (a) Phylogenetic analysis of CDs from *Tetrahymena* HP1 (Hhp1) with dHP1a, Pc, and human homologs. ClustalW alignment (MUSCLE) and the maximum-likelihood method (PhyML 3.0) (52) were used for tree generation, and the results were displayed by using TreeDyn 198.3 (58). The scale bar represents the number of amino acid substitutions per site. The branch length is proportional to the number of substitutions per site. Swi6 from *Schizosaccharomyces pombe* is included for evolutionary range. (b) Alignment of CD sequences shown in order of alignment with Hhp1. Green highlighting indicates residues that are important for Pc interactions with H3K27Me_3_ (9), and asterisks indicate those that distinguish Pc-type affinities for H3K27Me_3_ binding from HP1-like CDs (groups separated by the dashed line) (11). Light blue highlighting shows the aromatic cage residues responsible for methyl-lysine binding. The percentages of acidic and basic residues and the ratio of acidic to basic residues (a/b) in each CD are shown on the right. (c) Phylogenetic analysis of the entire CD protein sequences aligned in panel b. The scale bar represents the number of amino acid substitutions per site. The branch length is proportional to the number of substitutions per site.

The Hhp1 CD was first examined for the sequence features that distinguish H3K9 versus H3K27 methyl-lysine binding activities of HP1 and Pc family proteins that were previously identified through structural analyses of *Drosophila* and mammalian homologs (11, 12, 14). CD sequences from Hhp1, dHp1a, *Drosophila* Pc (dPc), human Cbx1-8, and the HP1 homolog Swi6 from fission yeast were aligned by using ClustalW. Human Cbx (chromobox) proteins were used because of their specific site differences that determine K9-methyl binding (HP1 homologs Cbx1, -3, and -5) or K27-methyl binding (Pc homologs Cbx2, -4, -6, -7, and -8) (14). We found that Hhp1 aligned most closely with other Pc-type CDs (Fig. 1a and b), and of these, most closely with mammalian Cbx6. In common with this group, Hhp1 possessed hydrophobic residues instead of acidic residues at methyl-lysine binding discrimination sites (14) (Fig. 1b, green highlights). Also in common with methyl-lysine 27 binding CDs, the Hhp1 CD had a less acidic character than that of methyl-lysine 9 binders (Fig. 1b), a feature proposed to permit K27-methyl binding (14). These results indicate that the CD of Hhp1 is structurally more similar to those on Pc-type proteins, which generally have higher binding affinities for H3 methyl-lysine 27 than those on HP1 family proteins. The commonality of Pc proteins is observed only in CD comparisons; alignments using full-length protein sequences group Hhp1 in the HP1 family (Fig. 1c), probably because of the presence of a CSD.

**FIG 2:**
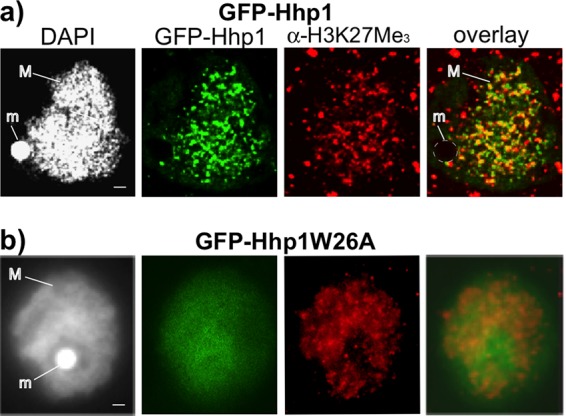
GFP-Hhp1 colocalizes with H3K27Me_3_. Cells expressing GFP-Hhp1 following induction with CdCl_2_ were fixed with paraformaldehyde, processed for immunofluorescence assay with anti-H3K27Me_3_ antiserum, and counterstained with DAPI. An image panel representing the signal distribution and degree of overlap observed in all imaged nuclei is shown. (a) Cells expressing wild-type GFP-Hhp1 imaged by confocal laser scanning fluorescence microscopy. (b) Cells expressing Hhp1W26A imaged by epifluorescence microscopy. M, macronucleus; m, micronucleus. Scale bars = 1 µm.

To explore whether Hhp1 recognizes the histone H3K27Me_3_ mark, colocalization was examined. Cells episomally expressing N-terminally fused green fluorescent protein (GFP)-Hhp1 were fixed in paraformaldehyde for detection by immunofluorescence assay with anti-H3K27Me_3_ antiserum. Consistent with previous studies, fluorescence microscopy revealed a punctate pattern of GFP-Hhp1 within the macronucleus, indicating Hhp1 concentration in the highly condensed chromatin bodies (22) (Fig. 2), whereas control cells episomally expressing GFP alone showed no macronuclear localization at any point in the cell cycle (see Fig. 6, GFP control). Similarly, the H3K27Me_3_ signal was observed in the expected punctate pattern because of its concentration in chromatin bodies (24) (Fig. 2a). The extranuclear signal from anti-H3K27Me_3_ varied between experiments and was considered background. The overlap of signals from both GFP-Hhp1 and anti-H3K27Me_3_ antiserum, estimated from cell nuclei in three separate experiments, supported Hhp1 colocalization with the histone H3K27Me_3_ mark. Fifty-two percent (±3%) of the GFP-Hhp1 concentration spots overlapped H3K27Me_3_ concentration spots, and 63% (±6%) of the H3K27Me_3_ concentration spots overlapped GFP-Hhp1 concentration spots. Previous work revealed that three “caging” amino acids within the CD are required for its interaction with methylated lysines on histone H3 (26). Tryptophan 26 on Hhp1 is one of these, and it lies within a highly conserved motif (**W**KG) on Pc homologs (14). We found that mutating tryptophan 26 to alanine (Hhp1W26A) caused Hhp1 to be delocalized from H3K27Me_3_ and, instead, be distributed more uniformly throughout the macronucleus (Fig. 2b). This demonstration that punctate colocalization of Hhp1 with H3K27Me_3_ was dependent on the presence of all caging amino acids was evidence that Hhp1 binds to the H3K27Me_3_ mark.

**FIG 3:**
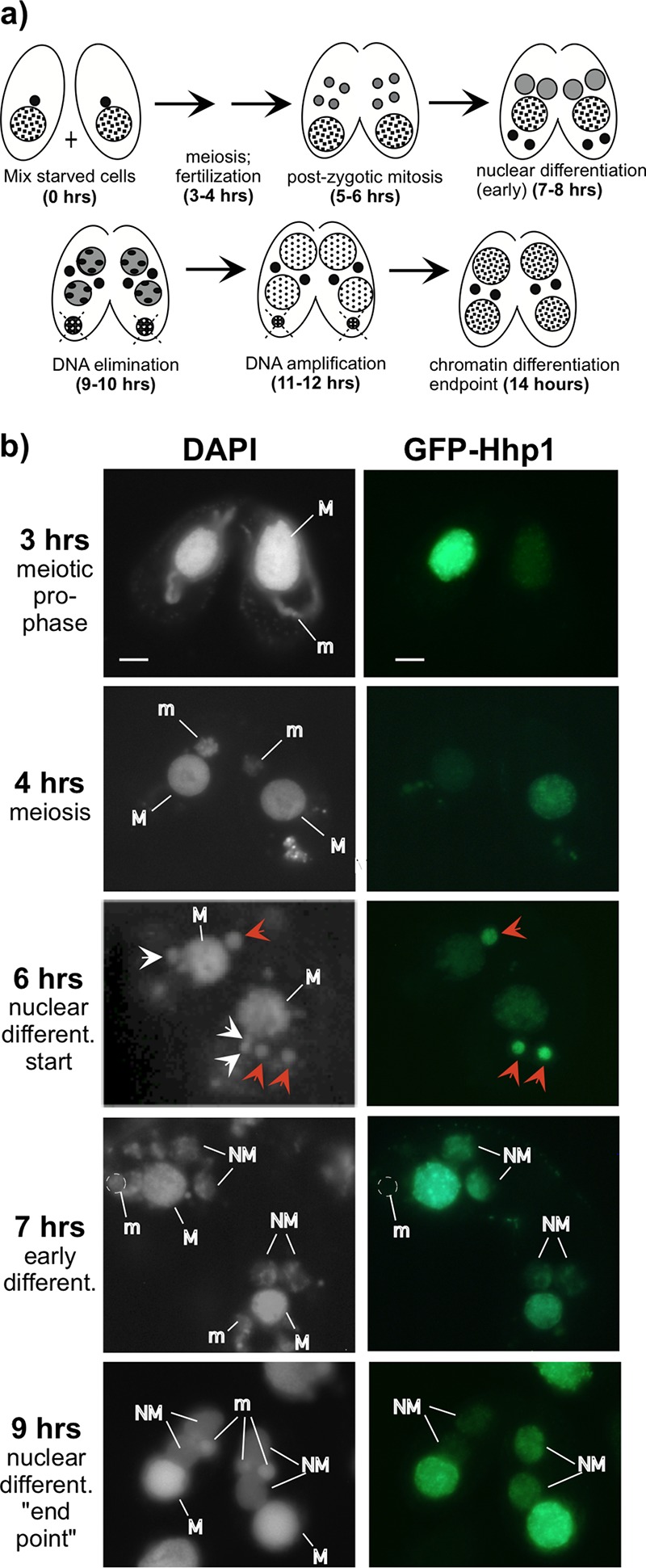
Hhp1 selectively localizes to nuclei differentiating into transcriptionally active macronuclei. (a) Schematic of nuclear development stages during *Tetrahymena* conjugation. Micronuclei, small black filled circles; macronucleus, large checkered circle. After postzygotic mitosis (5 to 6 h), two of the four resulting nuclei develop into new macronuclei (gray circles) while the other two develop into transcriptionally silent micronuclei (black filled). Some DNA is eliminated from new macronuclei (9 to 10 h), and the old macronucleus degrades. (b) Cells expressing GFP-tagged Hhp1 were mixed with cells of a different mating type. Conjugating cell samples taken at various time intervals were stained with DAPI, and live cells were visualized by fluorescence microscopy. M, macronucleus; m, micronucleus; NM, new macronucleus. White arrows indicate nuclei formed from postzygotic mitosis, and red arrows (6 h) indicate those selected for new macronucleus development (that migrate anterior to the macronucleus and are slightly larger) and that contain Hhp1. Only one of these is visible in one cell of the pair shown. The images shown represent the localization observed in all cell pairs observed at similar stages. Scale bar = 2 µm.

On the sexual conjugation pathway of *Tetrahymena*, nuclei undergo differentiation events that produce new transcriptionally silent micronuclei and transcriptionally active macronuclei (Fig. 3a). Given that Hhp1 is specific to the active macronucleus, we queried the point in the differentiation process at which Hhp1 appears in the differentiating nuclei, all of which possess chromatin with H3K27Me_3_ (24). Cells containing GFP-Hhp1 were starved and mixed with cells of another mating type, and expression of GFP-Hhp1 was induced with CdCl_2_ 2 h after mixing. GFP-Hhp1 was visualized by fluorescence microscopy at various time points in live cells in the conjugating cell population. GFP-Hhp1 localized only to the macronucleus and not to the decondensed (crescent) meiotic prophase micronucleus in early conjugation (h 3) or to the recondensed meiotic micronucleus at h 4 (Fig. 3b), ruling out a direct role in meiotic chromatin dynamics. Meiosis of the micronucleus produces gametes that cross-fertilize, and after fertilization, two successive mitotic divisions of the zygote produce four equivalent nuclei by h 6 (27). Two of these nuclei differentiate into transcriptionally silent, heterochromatinized micronuclei, and the other two differentiate into new transcriptionally active macronuclei, a process that involves genome amplifications and establishment of condensed and decondensed chromatin domains (Fig. 3a). Our analysis revealed that of the postzygotic nuclei, GFP-Hhp1 localized only to those destined for new macronucleus development at the initial selection point, when all postzygotic nuclei are similarly small, and remained only in those nuclei (in addition to the old macronucleus) throughout the rest of development (Fig. 3b, 6 to 9 h). This pattern suggested that Hhp1 participates in early events at the onset of the differentiation process, when domains of differentially condensed chromatin are first being established.

**FIG 4:**
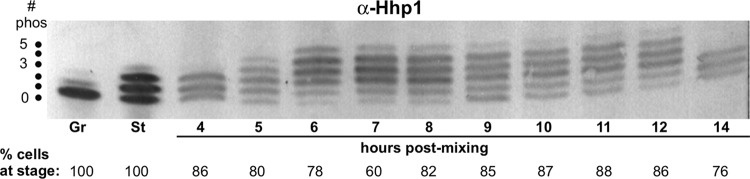
Hhp1 is multiply phosphorylated during nuclear differentiation and development. Shown is an immunoblot analysis of Hhp1 proteins throughout the course of conjugation and nuclear development. Total cellular proteins resolved on an acid urea-polyacrylamide gel were blotted and probed with anti-Hhp1 antiserum. Laddering is due to Hhp1 isoforms with various numbers of phosphoryl modifications shown on the left edge of the blot. The actual percentage of cells in each morphological stage corresponding to the hours shown in Fig. 3a is shown as the percentage of cells at that stage. # phos, number of phosphorylated sites; Gr, growing; St, starved.

To begin exploring requirements for Hhp1 localization, we focused on posttranslational modifications. Hhp1 was previously shown to be phosphorylated during vegetative growth and then more extensively when cells are starved (22). We extended this by surveying correlations between Hhp1 phospho isoforms and stages of conjugation and nuclear development, which might indicate potential roles for isoforms in modulating Hhp1 function. Cell conjugation was initiated, and synchrony of the developmental events was monitored by determining the fraction of conjugating pairs in a particular morphological stage through a series of measurements at 1-h intervals (Fig. 3a) (27). Whole-cell protein extracts made from the samples at each time point were resolved by size and charge through acid urea-polyacrylamide gel electrophoresis (AU-PAGE), and resolved Hhp1 isoforms were detected by immunoblotting with anti-Hhp1 antiserum. This method previously revealed distinct laddering of Hhp1 isoforms due to phosphorylation (23). Our analysis revealed an increase in Hhp1 modification during mitosis starting at h 5 in conjugation (Fig. 4). An additional isoform was observed immediately following postzygotic mitosis when Hhp1 first localizes to new differentiating macronuclei (6 h, Fig. 3a and 4). These densely modified isoforms were maintained throughout the rest of nuclear development, until the endpoint of development and chromatin differentiation at 14 h. The presence of highly modified Hhp1 isoforms correlated with major chromatin restructuring events occurring in the developing new macronuclei (establishment of euchromatin and heterochromatin domains and transcriptional activity) and in the old macronucleus (chromatin condensation and degradation) (Fig. 3a).

**FIG 5:**
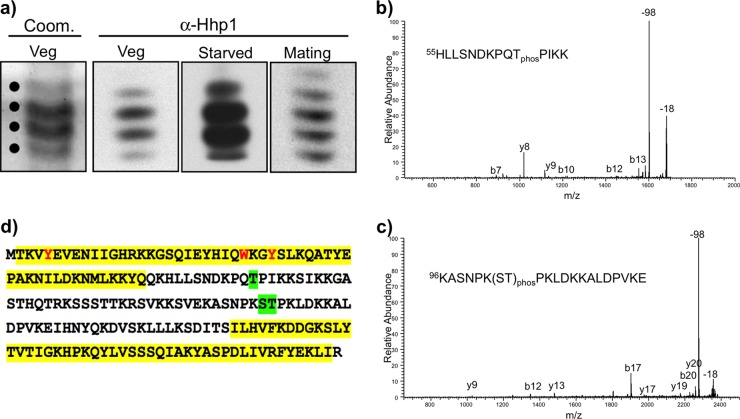
Identification of phosphorylation sites on Hhp1. (a) A majority of the Hhp1 isoforms were retained through the isolation procedure. Isoforms were resolved by AU-PAGE after partial purification by reversed-phase HPLC, and a portion of the sample was analyzed by immunoblotting with anti-Hhp1 antiserum. An example of Coomassie-stained bands that were excised for analysis by MALDI-MS/MS (indicated by dots) is shown for vegetatively growing cells (Coom. Veg). (b) Representative MALDI-MS/MS spectrum of an Hhp1 peptide phosphorylated on T64. The signature 98-Da loss for phosphopeptides is indicated, as well as the common 18-Da loss for peptides. Fragment ions are labeled. (c) Representative MALDI-MS/MS spectrum of an Hhp1 peptide phosphorylated at either serine 102 or threonine 103. (d) Amino acid sequence of Hhp1 showing phosphorylation sites that were identified on all Hhp1 isoforms: T64 and S102 or T103 (green highlighting). Yellow highlighting denotes the CD and CSD (the CSD is shortened by 17 amino acids at the N terminus from the original prediction [20] based on recent analysis with HHPRED [59]); methyl-lysine caging residues are red.

To query the role of specific phosphorylations, modification sites were first identified. Protein fractions enriched in Hhp1 from growing, starved, and 6-h conjugating cells were obtained by reversed-phase high-performance liquid chromatography (HPLC) and then resolved by AU-PAGE without substantial loss of Hhp1 isoforms (Fig. 5a). Bands representing the different isoforms of Hhp1 (confirmed by immunoblotting part of each fraction with anti-Hhp1 antiserum) were excised from the gel and analyzed by matrix-assisted laser desorption ionization–tandem mass spectrometry (MALDI-MS/MS) (28) to identify the sites of phosphorylation (Fig. 5a; Coom. Veg example). On average, 74% sequence coverage of Hhp1 was obtained for the isoforms analyzed. One phosphorylated site present in all populations of Hhp1 isoforms (mono-, di-, and trimodified, etc.) was identified as threonine 64 (T64) (Fig. 5b). A second phosphorylated site was present in all isoform populations, but only in vegetatively growing cells, i.e., serine 102 (S102) or threonine 103 (T103)—the analysis was unable to unambiguously resolve which of the latter two amino acids was preferentially modified (Fig. 5c). Figure 5d shows the locations of the sites of phosphorylation. T64 and T103 were previously predicted as phospho sites potentially recognized by Cdc2/Cdk1 kinase, which phosphorylated Hhp1 in *in vitro* experiments (22).

**FIG 6:**
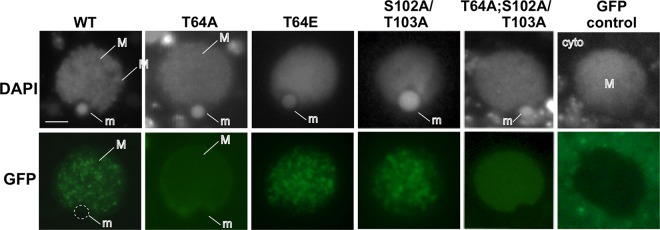
T64A mutation abolishes Hhp1 concentration in chromatin bodies. Growing cells expressing GFP-Hhp1 or GFP-tagged mutant versions of Hhp1 were induced with CdCl_2_ and stained with DAPI, and live cells were visualized by fluorescence microscopy. Only nuclei are shown. GFP control is expression of GFP alone (no fusion with Hhp1), which shows accumulation in the surrounding cytoplasm. WT, wild type; M, macronucleus; m, micronucleus; cyto, cytoplasm. Scale bar = 2 µm.

**FIG 7:**
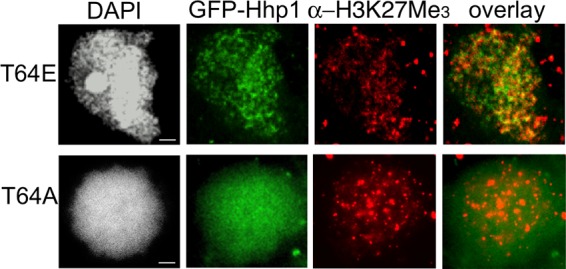
T64E mutation retains Hhp1 colocalization with H3K27Me_3_. Growing cells expressing GFP-Hhp1 or GFP-tagged mutant versions of Hhp1 were fixed with paraformaldehyde, subjected to immunodetection with anti-H3K27Me_3_ antiserum, counterstained with DAPI, and visualized by fluorescence microscopy. Scale bars = 1 µm.

To test whether phosphorylation at these two locations on Hhp1 influenced Hhp1 targeting to chromatin bodies within the macronucleus, the following site mutations of the Hhp1 gene were made: threonine 64 was changed to alanine (T64A) to abolish phosphorylation and changed to glutamic acid (T64E) to mimic phosphorylation. The same site mutations were made for S102/T10, as well as the combinations T64A;S102A/T103A and T64E;S102E/T103E. All Hhp1 mutants were N-terminally fused with GFP and episomally expressed in *Tetrahymena* cells, and live cells were imaged by fluorescence microscopy. As expected, wild-type Hhp1 concentrated in the punctate pattern of highly condensed chromatin bodies within the macronuclei of all of the cells examined (Fig. 6) (22). Eliminating phosphorylation of S102/T103 with S102A/T103A mutations did not alter the localization pattern from that produced by wild-type Hhp1 in all of the cells (>300) examined (Fig. 6). However, the pattern was abolished by the T64A mutation, either alone or in combination with S102A/T103A, which instead produced a weaker and more diffuse signal in all of the cells examined (>300). These results suggest that phosphorylation of T64 (but not S102/T103) is necessary for Hhp1 concentration in chromatin bodies. This idea is supported by the T64E phosphorylation mimic, which retained the wild-type punctate localization pattern in all of the cells observed. Colocalization experiments showed that the diffuse distribution of the T64A mutant form within the macronucleus was not due to any observable changes in H3K27Me_3_ distribution; the punctate concentration pattern was retained in all of the cells observed (>200) and in all 15 nuclei imaged (a representative image is shown in Fig. 7). Imaging also revealed that T64E retained colocalization with H3K27Me_3_ in all of the nuclei observed (>200) and in 3 nuclei quantified in three separate experiments (48% ± 3% overlap of anti-H3K27Me_3_ signal with GFP-Hhp1 signal; Fig. 7), suggesting that the phosphorylation mimic could recognize this mark to a degree similar to that of wild-type Hhp1 (Fig. 2). Together, the localization data indicate that phosphorylation of T64 is necessary for Hhp1 concentration in chromatin bodies marked with H3K27Me_3_.

## DISCUSSION

Compacting the genome into various chromatin structures is a highly dynamic process requiring precise spatial and temporal regulation. CD proteins that read posttranslational histone modifications are key players in the process, and a number of studies have revealed reversible posttranslational modifications that regulate the activity of these chromatin effectors. Toward the goal of understanding how these modifications mediate genome dynamics in response to external signals, learning how they affect the activity of chromatin proteins is a necessary step. To this end, the HP1 and Pc families have been a major focus. Our study is one of the first to explore the impact of posttranslational modification on HP1 homolog function in a protozoan system.

While *Tetrahymena* Hhp1 is a member of the HP1 family (with a CD, a CSD, and a serine/threonine-rich hinge region) we found that its CD has discriminating sequence features that are more similar to those found in Pc family proteins, which facilitate binding to trimethylated H3K27 (Fig. 1). This is consistent with the presence of H3K27 methylation and the complete lack of H3K9 methylation in *Tetrahymena* macronuclei where Hhp1 resides (24). In its colocalization with H3K27Me_3_ (Fig. 2), Hhp1 is similar to the HP1-like protein Tfl2/Lhp1 in *Arabidopsis*, which has a Pc-type CD with affinity for H3K27Me_3_ and localizes primarily to H3K27-methylated chromatin (29, 30). These two examples indicate that some HP1 CDs evolved greater affinity for the H3K27Me_3_ mark that is predominant on repressed chromatin in some organisms, and the *in vivo* colocalization that was dependent on methyl-lysine aromatic caging residues (Fig. 2) supports the model in which Hhp1 targets macronuclear chromatin bodies by directly binding H3K27Me_3_. Interestingly, this mark is also present in the micronuclei of cells, where Hhp1 is absent (23, 24). We speculate that this is due to lack of a specific micronuclear localization signal on Hhp1 that would permit its transport into the micronucleus; the micronucleus possesses nuclear pore complexes with components that are distinct from those on the macronucleus (31). Within the macronucleus, approximately 40% of the Hhp1 signal did not appear to colocalize with H3K27 methylation (Fig. 2). This result is consistent with recent genome-wide analyses that revealed methyllysine-independent binding of dHP1 paralogs to some chromatin regions (32). In our experiment, since a GFP signal marked the distribution of all modified and unmodified Hhp1 isoforms, and since T64 phosphorylation seems to be required for colocalization with H3K27Me_3_, it is expected that at least unmodified Hhp1 (~20 to 30% estimated from quantitation of anti-Hhp1 immunoblot assays of vegetative cells) and those monophosphorylated on S102/T103 would be distributed to other locales.

Hhp1 is necessary for starvation-induced chromatin condensation in *Tetrahymena* (22). Although the function of chromatin bodies is unknown, they fail to enlarge in cells lacking Hhp1 and this correlates with loss of expression of starvation-induced genes, suggesting a role for heterochromatin assembly via Hhp1 in gene induction. This observation is reminiscent of dHP1a, in which mutations decrease the expression of variegating heterochromatin-linked genes (33, 34). The present study assessed potential roles for Hhp1 during other periods in the life cycle involving chromatin reorganization. Hhp1 remained in the macronucleus throughout its programmed degradation in conjugating cells (Fig. 3b), a process that involves extensive chromatin condensation (35). We found no evidence of its participation in micronuclear decondensation or recondensation during meiosis but found that it selectively marked postzygotic nuclei slated for macronuclear development very early in the differentiation process. The presence of Hhp1 in early postzygotic nuclei suggests that it plays a role in the initial assembly of heterochromatin domains within a genome that is organizing for transcriptional activity. Given that all postzygotic nuclei possess H3K27Me_3_ at this stage (24), an interesting question is how Hhp1 is targeted specifically to the subset that is initiating transcriptional competency. The mechanism might involve posttranslational modifications of Hhp1 since an increase in differentially modified isoforms occurs at this time in development (Fig. 4). It is also possible that the more highly modified Hhp1 isoforms are arising in the old macronucleus instead of in the new macronucleus-destined postzygotic nuclei. With both possibilities, there is a correlation between the presence of highly modified isoforms and stages of development requiring major restructuring of chromatin, either in macronucleus-destined nuclei or in established macronuclear chromatin that is condensing and undergoing programmed nuclear death. Although the laddering of Hhp1 isoforms in our acid urea-gel analysis resembles that observed for phosphoryl modifications in previous work (22), we cannot rule out the possibility that laddering was caused in part by acetylation, which would show almost the same shift in isoform mobility in the gel system used (36).

This study revealed that singly and multiply modified Hhp1 isoforms from vegetatively growing, starved, and conjugating cells are phosphorylated at T64 (Fig. 5). T64 is located in the hinge region close to the CD and is a putative substrate for Cdc2 kinase, which was shown to phosphorylate Hhp1 *in vitro* (22). Our results indicate that phosphorylation of this site is necessary for Hhp1 targeting to highly condensed DNA in chromatin bodies marked with H3K27Me_3_ (Fig. 6 and 7). Interestingly, *Drosophila* Pc (dPc) and the mammalian Pc homolog Cbx7 are both phosphorylated and although the site(s) on dPc is unknown, on Cbx7, it occurs at the motif P*X*(**S/T**)P, which is highly conserved from flies to humans (37, 38). The T64 site on Hhp1 is also contained within the same motif, P*Q***T**P. In Cbx7, this motif is close to the Pc box sequence, which is essential for Cbx7 interaction with components of the Pc repressive complex (PRC1), and there is evidence that phosphorylation of this site via mitogen-activated protein kinase signaling enhances PRC1 interactions (37). Testing whether Hhp1 phosphorylation mediates interaction with other proteins in a similar way awaits the identification of interactors, which are currently unknown. Other studies implicate a reversible negative charge via phosphorylation on HP1 proteins in promoting the assembly and disassembly of complexes that regulate condensed chromatin domains during development (15) or during the regular cell cycle (39). For example, phosphorylation of HP1 proteins, primarily at the N and C termini, increased their association with methylated heterochromatin of flies (16), yeast (40), and mammals (18); increased their binding specificity for H3K9-methylated nucleosomes in mammals (19); and increased heterochromatin-mediated silencing in flies (15, 16). The best-characterized phosphorylation sites on these proteins lie within acidic NTEs adjacent to the CD, where phosphorylation is thought to strengthen the interaction of that region with the basic histone tails (18). Unlike these HP1 proteins, Hhp1 lacks an acidic NTE. We speculate that phosphorylation at T64 on the C-terminal side of the CD could enhance histone interactions by a similar charge-based mechanism. Another site (S102/T103) was also identified, but only on isoforms in vegetatively growing cells. This suggests that, in addition to the overall number of modifications on Hhp1, the physiological state also correlates with redistribution of kinase site usage. This could be more robustly addressed in future studies using technologies that detect modification combinations on single protein molecules.

Both the commonly phosphorylated T64 site and the S102/T103 site on Hhp1 reside in the hinge region. The hinge region of HP1 proteins is much less conserved across species, but despite this high sequence variability, it is emerging as a frequently phosphorylated region. One other HP1-like protein from *Tetrahymena*, Pdd1, was recently shown to be multiply phosphorylated in the hinge region in addition to the NTE (41, 42). Multiple classes of kinases are known to target hinge regions on homologs in different species, including casein kinase II (CKII), protein kinase A/CaCKII, protein tyrosine kinase, and Pim-1 kinase (40, 43 – 45). Our present work adds another to the list, Cdc2/Cdk1, which likely phosphorylates the sites we have identified on Hhp1 (22) and raises the possibility that binding to K27Me_3_-marked chromatin is regulated by the cell cycle. Prediction algorithms identified other high-probability kinase consensus sites, most concentrated in the serine/threonine-rich hinge region of Hhp1 (data not shown). These could account for the highly modified isoforms detected in this study (up to five modifications), but they but were not identified as phospho sites by our MALDI-MS/MS approach. Analysis of the sequence coverage revealed that peptides mapping to the hinge region containing these other consensus phosphorylation sites were underrepresented in or absent from our MALDI-fragmented samples (data not shown).

The concentration and variability of kinase sites in HP1 hinge regions raise possibilities for diversifying functions of HP1 paralogs in response to signaling, especially given that the functional outcomes of hinge phosphorylation vary. On dHP1, mutating hinge consensus phosphorylation sites to alanine or glutamic acid reduced or abolished heterochromatin-mediated silencing and suggested that a balance of hyper- and hypophosphorylated HP1 is necessary for normal levels of silencing (45). Consistent with this idea, another study showed that simultaneously abolishing multiple hinge phosphorylations on dHP1 increased silencing above normal levels (46). Similar mutations of CKII sites on the human HP1β hinge produced no effect on chromatin binding (20), but loss of CKII phosphorylation on the hinge of Swi6 in fission yeast reduced heterochromatin silencing. Other mechanistic insights into the hinge-mediated chromatin function of HP1 have also emerged. The hinge region alone from dHP1 and *Xenopus* HP1 was able to localize to H3K9Me chromatin (44, 47) and also to bind DNA (47, 48), a mechanism that may involve additional interactions with RNA. Hinge region interactions with RNA were found to be necessary for dHP1 pericentric localization (49), in combination with the correct histone modifications (H3K9Me_3_). Posttranslational modifications of HP1 may serve to regulate these interactions with other factors. In addition to phosphorylation, other modifications found within the hinge regions of some HP1 homologs, including sumoylation and acetylation, are speculated to regulate interactions with DNA, RNA, and proteins such as components of the ORC complex (reference 4 and papers cited there). In general, the variation in the length and sequence of the hinge regions is thought to be an important source of functional differentiation of HP1 family proteins (49), perhaps through variability in these modification sites that regulate chromatin targeting and function. Our study revealed one such modification that affects the properties of Hhp1 in *Tetrahymena*. As most studies on CD protein modifications to date have been done with yeast, mammals, and *Drosophila*, revelations about evolutionarily conserved modification patterns and their biological significance will come through similar studies with a greater diversity of organisms.

## MATERIALS AND METHODS

### Bioinformatics.

The amino acid sequences of selected CD proteins were analyzed by using BLASTP (http://www.ncbi.nlm.nih.gov) and Pfam (http://pfam.xfam.org) to determine the CD boundaries of each. Alignment comparisons of the CD sequences were performed with Multiple Sequence Alignment-ClustalW (EMBL-EBI, Welcome Trust Genome Campus, Cambridgeshire, United Kingdom; http://www.ebi.ac.uk/Tools/msa/clustalw2/). The CLUSTAL protein sequence alignment was performed by using a gap-opening penalty of 10, a gap extension penalty of 0.05, a hydrophobic gap, no weight transition, and a BLOSUM weight matrix. Molecular phylogenetic relationships were computed by first aligning sequences by multiple sequence comparison by log expectation (MUSCLE) by using default parameters (50). The output, in the Pearson/FASTA format, was analyzed by using the maximum-likelihood method (PhyML 3.0; http://phylogeny.lirmm.fr/phylo_cgi/index.cgi) with the EX2 substitution model (51, 52). Branch support was computed by using SH-like approximate likelihood ratio tests.

### Strains and cell culture conditions.

*T. thermophila* strains CU427 (*chx1-1/chx1-1 CHX1*; cy-s, VI) and CU428 (*mpr1-1/mpr1-1 MPR1*; mp-s, VII), provided by the national *Tetrahymena* Stock Center at Cornell University, were used as wild-type strains. For all experiments, *T. thermophila* strains, including the strain expressing GFP-Hhp1 and mutant variants, were grown in 2% PPYS medium (0.02 g/ml proteose peptone, 0.002 g/ml yeast extract, 0.03 mg/ml sequestrine) containing 2× PSF (penicillin, streptomycin, and amphotericin B [Fungizone]; Gibco-BRL) with shaking (100 rpm) at 30°C until mid-logarithmic phase (1 × 10^5^ to 3 × 10^5^ cells/ml). For cell starvation, cells were washed once and suspended in 10 mM Tris-HCl (pH 7.4) at a density of 3 × 10^5^/ml and then incubated for 15 to 18 h at 30°C without shaking. To conjugate cells, starved cells (CU427, CU428, or strains expressing GFP-Hhp1 or mutant variants) were mixed in equal numbers in petri dishes and incubated at 30°C without shaking in a moist chamber.

To induce expression of GFP-Hhp1 or GFP-Hhp1 mutant variants, CdCl_2_ was added to growing cells (final concentration of 2 µg/ml) or to starved/conjugating cells (final concentration of 0.2 µg/ml) and incubated for 2 h before visualization by fluorescence microscopy.

### Plasmid construction.

The Hhp1 gene was amplified from *Tetrahymena* genomic DNA by PCR with forward primer 5′ CACCATGACAAAAGTTTACGAAGTAGAA 3′ and reverse primer 5′ TCAGCGGATTAGCTTTTATAGAATC 3′. The resulting PCR product was directionally cloned into plasmid pENTR/D-TOPO (Invitrogen) to make plasmid pENTR-*HHP1*, which was transformed into chemically competent *Escherichia coli* TOP10 cells (Invitrogen). The cloned *HHP1* sequence was first verified by Sanger sequencing (with primers M13-F and M13-R) and then recombined with the Gateway cassette in pIGF-GTW (31) by combining 150 ng of pENTR-*HHP1* (entry clone), 400 ng of pIGF-GTW, and 1 µl of recombinase enzyme (LR Clonase II; Invitrogen) and incubating the reaction mixture for 20 h at 22°C. The Gateway recombination reaction products were electroporated into electrocompetent *E. coli* DH10B (prepared as previously described [53]). The resulting pIGF-GTW::*HHP1* plasmid contained the *HHP1* gene fused at its amino terminus to GFP under the control of the *MTT1* promoter. The fusion construct was confirmed by Sanger sequencing. Mutagenesis of *HHP1* was performed on pENTR-*HHP1* with the QuikChange Lightning site-directed mutagenesis kit (Agilent Technologies) in accordance with the manufacturer’s directions and confirmed by Sanger sequencing prior to recombination onto pIGF-GTW as described above.

### *Tetrahymena* transformation, induction, and expression analysis.

The pIGF-GTW::*HHP1* construct for N-terminal GFP fusion of *HHP1* was transformed into conjugating CU428 × CU427 strains by electroporation at 9, 9.5, and 10 h into conjugation according to a previously published method (54). Transformants were selected by growth in 100 µg/ml paromomycin. Expression of the fusion proteins was induced by incubating cultures of *Tetrahymena* transformants for 2 h in CdCl_2_ (2 µg/ml for growing cells; 0.2 µg/ml for starved and conjugating cells). Expression of the fusion proteins was confirmed by immunoblotting with anti-GFP antiserum (1:1,000 in Tris-buffered saline [TBS]–1% milk), GF28R; Aviva Systems Biology catalog number OAEA00007).

### AU-PAGE and immunoblot analysis.

Whole-cell protein lysates were generated from 1 × 10^7^ cells of strain CU428 (wild type). Cells were pelleted by centrifugation at 4,000 × *g* for 2 min, lysed by resuspension in 100 µl of whole-cell lysis buffer (50 mM Tris [pH 6.8], 1.5% SDS, 7.5% β-mercaptoethanol, 0.75 mM phenylmethylsulfonyl fluoride), immediately boiled for 3 min, and then flash-frozen in a dry ice-ethanol bath. Fifteen microliters of acid urea-gel loading dye was added to 30 µl of cell lysate, and samples were resolved by electrophoresis for 22 h on a 30-cm acid urea-polyacrylamide gel by a previously described method (36). Resolved proteins were transferred to nitrocellulose membrane, blocked in 5% milk in TBS, and incubated with anti-Hhp1 antiserum (gift from C. D. Allis) diluted 1:2,000 in 1% milk–TBS–0.1% Tween 20. The membrane was washed three times for 5 min in TBS, incubated with alkaline phosphatase-conjugated goat anti-rabbit antiserum (Pierce 31340) diluted 1:5,000 with 1% milk in TBS–0.1% Tween 20, and developed with 5-bromo-4-chloro-3-indolyl-phosphate (BCIP)–nitroblue tetrazolium in accordance with the manufacturer’s directions (Promega vector S3771).

To confirm the identities of putative Hhp1 bands stained with Coomassie blue, 1/100 of the reversed-phase HPLC fraction enriched in Hhp1 (see Hhp1 isolation) was resolved by AU-PAGE and processed for immunoblotting as described above. The primary anti-Hhp1 antibody was diluted 1:2,000 in 1% milk–TBS–0.1% Tween 20. Secondary detection was done with goat anti-rabbit IgG (Jackson ImmunoResearch) diluted 1:5,000 in 1% milk–TBS–0.1% Tween 20.

### Indirect immunofluorescence and microscopy.

Growing and conjugating cells were fixed in 2% paraformaldehyde and processed for immunofluorescence assay as previously described (27). Fixed cells were incubated overnight at 4°C with anti-H3K27Me_3_ antiserum (1:500; Active Motif catalog number 39155) in 1% bovine serum albumin (BSA)–phosphate-buffered saline (PBS)–0.1% Tween 20 (PBST). Cells were then washed three times for 10 min in PB, incubated in secondary antiserum (rhodamine-conjugated goat anti-rabbit IgG, 1:100; Jackson ImmunoResearch catalog number 111-025-003) in PBST for 1 h at 37°C, and washed three times for 15 min in PBS. Fixed cells were counterstained by incubation with 0.1 µg/ml 4ʹ,6-diamidino-2-phenylindole (DAPI) in 0.1% BSA–PBS for 10 min. Cells were mounted by adding 5 µl of Vectashield Mounting Medium (Vector Laboratories Inc.) to the cell surface before a coverslip was laid over the sample.

For all live-cell GFP imaging, 1 × 10^5^ to 3 × 10^5^ cells in culture were centrifuged at 2,000 × *g* and the pellet was incubated for 5 min with 0.1 µg/ml DAPI (Sigma Chemicals). One microliter of the concentrated cells was added to 5 µl of 2% methylcellulose on a microscope slide and covered with a number 1.5 micro coverslip. Epifluorescence imaging was performed on a Leica DM4000 B LED fluorescence microscope at ×100 magnification, and confocal imaging was performed on a Leica DMi8 confocal fluorescence microscope.

For colocalization studies, GFP and rhodamine signals were imaged separately on fixed samples (described above) and overlaid with Leica Application Suite v4.3 software. Signal overlap in the macronucleus was quantified by using GFP images, rhodamine images, and GFP-rhodamine overlaid images of three different nuclei. Each image was divided into four circular areas, and each circle was divided into quadrants, for a total of 16 quadrants per image. The spots of GFP concentration in each quadrant were counted. Similarly, the spots of rhodamine concentration were counted. The number of rhodamine spots that overlapped GFP spots was then obtained from the overlaid images. By using these numbers, the fraction of GFP concentration that overlapped rhodamine concentration, and vice versa, was calculated.

### Hhp1 isolation and analysis by MS.

The total nuclei of 10^8^ vegetative, starved, and conjugating (6.5 h) cells were isolated by differential centrifugation as previously described (55, 56). Nuclei were acid extracted, and solubilized nuclear and chromatin-binding proteins were recovered by precipitation with trichloroacetic acid as previously described (36). Proteins in each sample were fractionated by reversed-phase HPLC with a C_8_ reversed-phase column. Fractions containing Hhp1 were identified by immunoblotting with anti-Hhp1 antiserum (see immunoblot analysis), pooled, resolved by AU-PAGE, and then visualized by staining with Coomassie brilliant blue R-250. The protein bands corresponding to the isoforms observed under vegetative, starved, and conjugating conditions were excised, destained, and subjected to in-gel proteolysis with trypsin or GluC. Resulting peptides were crystallized in 2,5-dihydroxybenzoic acid for MALDI-MS/MS analysis (57). Hypothesis-driven multiple-stage MS was used to specifically look for phosphorylation of serine or threonine residues as previously reported (28). Briefly, *m/z* values for all of the theoretically phosphorylated peptides from Hhp1 were calculated. These calculated *m*/*z* values were screened for the signature loss of 98 Da that is observed in the tandem mass spectrum of phosphorylated peptides. The presence of the 98-Da loss and other fragment ions (in both MS/MS and three-stage MS) were used to determine the presence of a phosphopeptide. Spectra were manually interpreted.
